# Effects of Selenium- and Zinc-Enriched *Lactobacillus plantarum* SeZi on Antioxidant Capacities and Gut Microbiome in an ICR Mouse Model

**DOI:** 10.3390/antiox9101028

**Published:** 2020-10-21

**Authors:** Sini Kang, Rui Li, Hui Jin, Hyun Ju You, Geun Eog Ji

**Affiliations:** 1 Department of Food and Nutrition, Research Institute of Human Ecology, Seoul National University, Seoul 08826, Korea; kangsini@snu.ac.kr (S.K.); lirui@ribolia.com (R.L.); huijin1030@hotmail.com (H.J.); 2 Institute of Health and Environment, Graduate School of Public Health, Seoul National University, Seoul 08826, Korea; 3 Research Center, BIFIDO Co., Ltd., Hongcheon 25117, Korea

**Keywords:** selenium, zinc, bioaccumulation, antioxidant capacities, gut microbiota

## Abstract

Selenium and zinc are essential trace minerals for humans with various biological functions. In this study, selenium- and zinc-tolerant lactic acid bacteria (LAB) isolates were screened out from human fecal samples. Amongst three hundred LAB isolates, the *Lactobacillus plantarum* SeZi strain displayed the tolerance against selenium and zinc with the greatest biomass production and bioaccumulation of selenium and zinc. To further assess the characteristics of this strain, the lyophilized *L. plantarum* SeZi were prepared and administered to Institute of Cancer Research (ICR) mice. The mice were divided into four groups, provided with normal chow (Con), or normal chow supplemented with Na_2_SeO_3_ and ZnSO_4_∙7H_2_O (SZ), *L. plantarum* SeZi (Lp), or selenium- and zinc-enriched *L. plantarum* SeZi (SZ + Lp), respectively. After 4 weeks of oral administration, the concentrations of selenium and zinc in blood were significantly increased in the SZ + Lp group when compared to the control or SZ group (*p* < 0.05). The increased selenium level led to an enhanced glutathione peroxidase activity and decreased blood malondialdehyde level in the SZ + Lp group (*p* < 0.05). Meanwhile, the results of bacterial community and microbial metabolic pathway analysis via 16S rRNA gene amplicon sequencing showed that *L. plantarum* SeZi significantly promoted the utilization of selenocysteine, seleno-cystathionine and seleno-methionine in the selenocompounds metabolism. Here, the in vivo antioxidant capacities of the selenium- and zinc-enriched lactobacillus strain showed us the utilization of a unique probiotic as a Se/Zn supplement with high availability, low toxicity, and additional probiotic advantages.

## 1. Introduction

Micronutrient deficiencies, known as “hidden hunger”, have affected more than 50% of the world’s population [1]. Selenium (Se) is a vital trace element, contributing to modulation of growth, regulation of antiviral capacity, and prevention of disease, especially cancer and cardiovascular disease [2,3,4]. The antioxidant activity of selenium is exhibited as a form of selenoenzymes, including glutathione peroxidase (GSH-Px), selenoprotein P, thioredoxin reductase, and methionine sulfoxide reductase [5]. Selenium deficiency can trigger serious health issues such as poor growth, muscle pain, decreased immune responses, and hypofunction of glandula thyreoidea [6,7]. Besides, the deficit of selenium is associated with a cardiomyopathy named as Keshan disease (KD) and an osteoarthropathy named as Kashin-Beck disease (KBD) [8]. Although KD and KBD are just local problems primarily in China and east Serbia due to environmentally low selenium [9], the hypothyreose or insuline secretion impairments associated with the lack of selenium have a global impact. Zinc (Zn) is another essential micronutrient for humans. It is a key component of many metalloenzymes (i.e., superoxide dismutase (SOD), carbonic anhydrase, alcohol dehydrogenase) associated with human growth, immunity, fertility, and reproduction [10]. Additionally, zinc is significant for the correct secretion of hormone isoline by pancreas [11]. The chronic deficiency of zinc can lead to glucose intolerance and pre-diabetic syndromes [12,13]. On the other hand, zinc chronic overdose, which can be caused by some nutraceuticals, might be responsible for some neurodegenerations such as nervus opticus inflammation [14,15].

Inorganic selenium, such as selenate (SeO_4_^2−^) and selenite (SeO_3_^2−^), are toxic and poorly bioavailable [16]. The reduction of selenium oxyanions largely depends on biotic processes by microorganisms [17]. The utilization of microorganisms as the natural adsorbent for metal ions (i.e., selenium and zinc) is eco-friendly and cost-effective [18]. The bioabsorption capacities are attributed to their intrinsic biochemical and structural properties of the cellular membrane [19]. Lactic acid bacteria (LAB), as important food-grade bacteria with probiotic advantages, have been extensively studied in this field. The selenium concentration in the medium is highly linked to bacterial selenium level, but the growth of most bacterial isolates from the human gut can be inhibited by the addition of inorganic selenium into the medium [20]. However, some LAB strains have been reported to be capable of resisting selenium oxyanions at high concentrations during cultivation [21,22,23]. Especially, *Lactobacillus plantarum* has been suggested as Se-enriched lactobacilli for food applications [24]. Few studies about Zn-enriched LAB have been conducted, but it has been found that the bacterial growth and probiotic effect of *L. plantarum* can be enhanced by zinc in the gut [25]. 

Although the resistances of LAB to selenium and zinc have been reported, the in vivo antioxidant capacities of the SeZn-enriched probiotic products have not been reported. In this study, SeZn-tolerant LAB strains isolated from human feces were screened out to further investigate the effects on selenium and zinc bioaccumulation and related metabolism, antioxidant activities, and compositional changes of intestinal microbiota in vivo in an Institute of Cancer Research (ICR) mouse model. 

## 2. Materials and Methods 

### 2.1. Isolation of Probiotic Strains from Human Feces

According to the protocol approved by the Institutional Review Board of Seoul National University (IRB No. 1702/002-013), fresh fecal samples were obtained from five children (1–6 years old) in Korea and stored at 4 °C during transportation. Each fecal sample (1 g) was serially diluted with a sterilized phosphate buffered saline solution (pH 7.4). The suspension was plated on *Lactobacillus* Selection (LBS) agar (Difco, Sparks, MD, USA) to isolate *Lactobacillus* spp. The plates were incubated anaerobically at 37 °C for 48 h [26]. Three hundreds of morphologically different microbial colonies were collected and cultured for further tests. The isolated LAB strains were then cultured in De Man, Rogosa and Sharpe (MRS) medium (Becton Dickinson, Cockeysville, MD, USA) containing 0.05% L-cysteine hydrochloride anaerobically at 37 °C. The bacterial stocks were stored at −80 °C with 17% glycerol utilized as a cryoprotectant.

### 2.2. Screening of SeZn-Tolerant LAB Isolates from Human Feces

To identify selenium-tolerant strains, the isolates were plated on the MRS agar in the presence of 60 mM Na_2_SeO_3_ (Sigma-Aldrich, St. Louis, MO, USA) at 37 °C for 24 h under anaerobic condition. When the concentration of selenium in the medium is high, strains convert inorganic selenium into element of selenium (red color) in the medium [27]. Thus, the strains with selenium resistance were selected based on the results of bacteria growth and color changes. 

Thereafter, the screened strains were further tested for zinc-tolerant abilities by culturing in the MRS agar with 100 mM ZnSO_4_·7H_2_O (Sigma-Aldrich) at 37 °C for 24 h under anaerobic condition. Strains with strong zinc tolerance were selected by observing the bacterial growth. The final screened SeZn-tolerant bacteria were identified by phylogenetic analysis of 16S rRNA gene sequence.

### 2.3. Assessing Bioaccumulation of Selenium and Zinc in LAB Strains During the Cultivation

Considering the application for food and feed additive, the initial concentrations of Na_2_SeO_3_ and ZnSO_4_·7H_2_O were set at 0.01 mM and 3.5 mM, respectively. After 24 h anaerobic culture in MRS broth, the LAB strains were centrifuged (15,600× *g*, 5 min) to gain pellets. The bacterial pellets were washed three times with phosphate buffered saline (PBS) and frozen at −80 °C for lyophilization. One liter of PBS buffer (pH 7.4) was prepared by dissolving NaCl (8 g), KCl (200 mg), Na_2_HPO_4_ (1.44 g) and KH_2_PO_4_ (245 mg) in the distilled water and autoclaved at 121 °C for 15 min.

The concentrations of zinc and selenium in the bacterial biomass were measured using an inductively coupled plasma-atomic emission spectrometer (ICP-AES, Optima-4300 DV, Perkin Elmer, Waltham, MA, USA). The lyophilized sample (400 mg) was digested with HNO_3_ (5 mL) and HClO_4_ (0.5 mL) by heating in a Multiwave 3000 microwave. After the cool-down to room temperature, the solution was diluted with deionized water to reach a final volume of 20 mL, and mineral levels were assessed by the Inductively coupled plasma atomic emission (ICP-AES). The bioconversion rates of Se and Zn were calculated by dividing the Se or Zn content in dry cell mass by the total Se or Zn content added in the broth. The strain with the highest levels of selenium and zinc bioaccumulation was selected as the experimental strain for in vivo study.

Bioconversion rate of Se (%) = (Se content in dry cell mass/total Se content added in broth) × 100%

Bioconversion rate of Zn (%) = (Zn content in dry cell mass/total Zn content added in broth) × 100%

### 2.4. Gene Analysis of Se/Zn Uptake and Resistance in L. plantarum SeZi

The genomic DNA of pure cultured *L. plantarum* SeZi isolate was extracted by using MG™ Cell Genomic DNA Extraction SV kit (MGmed, Seoul, Korea), following the manufacturer’s instructions. Whole genome sequencing was carried out by using a Nextera XT Library Preparation kit (Illumina, San Diego, CA, USA) and sequenced at a read length of 300 bp with paired-end library via an Illumina MiSeq sequencer (Illumina, San Diego, CA, USA). The Illumina sequencing raw data in the FASTQ format were assembled with SPAdes 3.9.0. Gene-finding and functional annotation pipeline of whole genome assemblies used in EzBioCloud genome database (http://www.ezbiocloud.net, ChunLab Co., Ltd., Seoul, Korea) [28]. The tRNA genes were investigated via tRNAscan-SE 1.3.1 [29]. The rRNA and other non-coding RNAs were explored by using a Rfam covariance model version 12.0 [30]. Protein-coding sequences (CDSs) were predicted via Prodigal 2.6.2 [31], and classified into different functional groups (EggNOG 4.5; http://eggnogdb.embl.de) [32]. In order to obtain more functional annotation, the UBLAST program [33] was utilized to search and compare the predicted CDSs in the protein databases, including Swissprot [34], Kyoto Encyclopedia of Genes and Genomes (KEGG) [35] and subsystems-based annotations (SEED) [36]. The comparative genomics analysis was conducted by using the genome sequences of closely related *Lactobacillus plantarum* strains from the EzBioCloud database and analyzed via ChunLab’s comparative genomics tool (http://www.ezbiocloud.net/contents/cg).

### 2.5. Effect of SeZn-Enriched L. plantarum SeZi in an ICR Mouse Model

#### 2.5.1. Preparation of SeZn-Enriched *L. plantarum* SeZi for Mouse Study 

The *L. plantarum* SeZi strain was anaerobically grown at 37 °C for 24 h in MRS medium with the addition of 0.01 mM Na_2_SeO_3_ and 3.5 mM ZnSO_4_∙7H_2_O. For harvesting probiotic powder, the bacterial pellets were collected by centrifugation (15,600× *g*, 5 min) after SeZn enrichment, thoroughly washed with the PBS buffer, and frozen at −80 °C for lyophilization.

#### 2.5.2. Animals and Diets

Seven-week old male ICR mice were purchased from Central Lab Animal (Seoul, Korea). The animal breeding environment was adjusted to a dark cycle of 12 h light/12 h dark at a temperature of 23 ± 1 °C and a humidity of 40–60%. The mice were acclimatized in the laboratory room for one week and then randomly divided into four groups (*n* = 8/group). The control group was provided with a normal chow diet AIN-93G purchased from Doo Yeol Biotech (Seoul, Korea). The treatment groups (SZ, Lp, SZ + Lp) were fed the same normal chow diet mixed with Na_2_SeO_3_ (1.2 µg/g Se^4+^) and ZnSO_4_∙7H_2_O (5 µg/g Zn^2+^), 10^12^ CFU/mouse *Lactobacillus plantarum* SeZi, and 10^12^ CFU/mouse SeZn-enriched *L. plantarum* SeZi, respectively. The daily administration was conducted for 4 weeks. The protocols and facilities utilized in this animal experiment were approved by the Institutional Animal Care and Use Committee of Seoul National University (SNU-180403-2-2).

#### 2.5.3. Blood Analysis

To assess Se and Zn concentrations and oxidative stress-related parameters in mouse blood, blood samples were collected into a 1.5 mL heparinized tube from the mouse heart by cardiac puncture. Approximately 0.9 mL of the blood samples were centrifuged (2500 rpm, 10 min) to separate serum. The serum and whole blood samples were stored at −80 °C. The concentrations of selenium and zinc in the whole blood were measured using ICP-AES. GSH-Px activity, SOD activity, and malondialdehyde (MDA) level in serum were assessed via antioxidant enzyme detection kits purchased from Jiancheng Bioengineering Institute, Nanjing, China.

#### 2.5.4. Bacterial Community Analysis by 16S rRNA Gene Amplicon Sequencing

Fecal DNA was extracted using a QIAamp DNA Stool Mini Kit (Qiagen, Manchester, UK). The V3-V4 hypervariable regions of the 16S rRNA genes in the stool DNA samples were targeted and amplified using interest-specific primers. A pooled library was constituted by attaching specific barcode sequences to the 16S rRNA amplicons. The denatured and diluted pooled library and PhiX control (Phix control v3, 30%, *v*/*v*) library were mixed and loaded onto a MiSeq v2 (500 cycle) reagent cartridge (Illumina, San Diego, CA, USA). The primers and methods were as described in our previous study [37]. After the metagenomic sequencing, paired-end FASTQ files were collected and imported into Quantitative Insights Into Microbial Ecology 2 (QIIME2) (ver. 2020.6, https://qiime2.org) for analysis. Operational taxonomic unit (OTU) taxonomy and related analysis were performed using QIIME2 as described in our previous study [37]. KEGG associated with selenocompounds metabolism pathways were assessed by conducting phylogenetic investigation of the community by reconstruction of unobserved states (PICRUSt) with the entire picrust2 pipeline command [38].

### 2.6. Statistic Analysis

Differential abundance analyses were performed by non-parametric one-way analysis of variance (ANOVA) using the Kruskal-Wallis test, or non-parametric *t*-test with Mann-Whitney test. Other analyses were conducted by one-way ANOVA with Tukey’s multiple comparisons test or paired *t*-test analysis. All statistical analyses were carried out via Graph-Pad Prism 8. Statistically significant difference was accepted at *p* < 0.05.

## 3. Results

### 3.1. Screening and Selection of SeZn-Tolerant LAB Strains

Amongst the three hundreds of isolated strains, only four LAB species grew in the presence of 60 mM selenite, including *L. plantarum, L. pentosus, L. fermentum,* and *L. rhamnosus*. All of these four strains are able to resist 100 mM ZnSO_4_·7H_2_O. Among these four SeZn-tolerant strains, *L. plantarum* SeZi yielded the greatest dry cell mass with the best selenium and zinc bioaccumulation capability as shown in Table 1. Based on the results of whole genome sequencing analysis in Table 2 and Table 3, the genes coding DedA and CysA proteins related to Na_2_SeO_3_ uptake and detoxification, and zinc uptake regulation protein ZUR (zinc uptake regulation) and zinc resistance protein MerR (Mercury resistance) were found in *L. plantarum* SeZi genome. Thus, *L. plantarum* SeZi was selected for in vivo mouse study. The abundances of these microbial selenium/zinc metabolism-related genes in genomes from other LAB strains were investigated using publicly deposited genome databases (NCBI genome datasets, https://www.ncbi.nlm.nih.gov/genome/). Among the 1912 genome assemblies available, only a few *Lactobacillus* genomes contain genes encoding DedA, CysA, ZUR, or MerR proteins (Table 4).

### 3.2. Increased Concentrations of Selenium and Zinc in Blood after L. plantarum SeZi Administration

**Figure 1:**
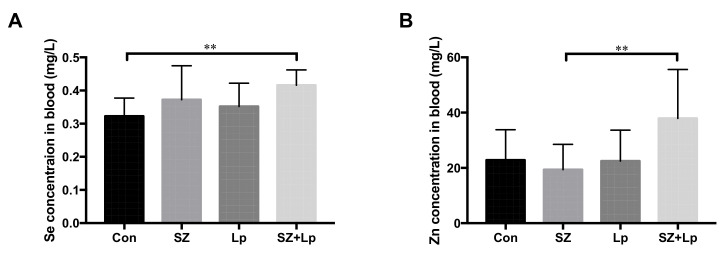
The concentrations of blood selenium (**A**) and zinc (**B**) in mice after 4 weeks of administration. Data were analyzed by unpaired *t*-test analysis and expressed as mean ± SD. ** *p* < 0.01 (*n* = 8). Con, control; SZ, selenium and zinc supplemented; Lp, *Lactobacillus plantarum* SeZi; SZ + Lp, selenium- and zinc-enriched *L. plantarum* SeZi.

The selenium and zinc contents in mouse blood are presented in Figure 1. The blood selenium and zinc levels in the SZ + Lp group were significantly higher than that of the control group and the SZ group, respectively (*p* < 0.05). The differences between the control, SZ and Lp groups were not significant (*p* > 0.05).

### 3.3. Increased Antioxidant Activities in Mice after L. plantarum SeZi Administration

**Figure 2:**
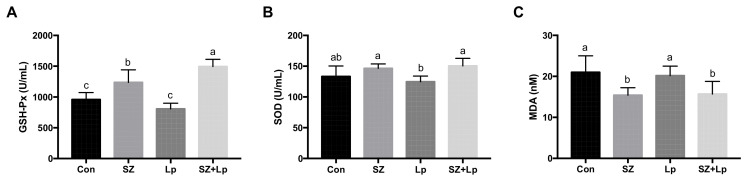
Glutathione peroxidase (GSH-Px) activity (**A**), superoxide dismutase (SOD) activity (**B**), and lipid oxidation product malondialdehyde (MDA) level (**C**) in serum of mice at the final day. Treatments with different letters (a, b, c) are significantly different at *p* < 0.05 (*n* = 8). Con, control; SZ, selenium and zinc supplemented; Lp, *Lactobacillus plantarum* SeZi; SZ + Lp, selenium- and zinc-enriched *L. plantarum* SeZi.

GSH-Px and SOD are imperative antioxidant defenses against oxidative stress [39,40], and MDA is the most commonly utilized biomarker of oxidative stress [41]. As shown in Figure 2, the GSH-Px activity was highest in the SZ + Lp group, followed by the SZ group when compared with other groups (*p* < 0.05). The activity of SOD was significantly increased in the SZ + Lp group compared to the Lp group (*p* < 0.05). Meanwhile, significant decreases in the MDA level were observed in the SZ group and SZ + Lp group compared to other two groups (*p* < 0.05).

### 3.4. Changes in the Gut Microbiota after L. plantarum SeZi Administration

**Figure 3:**
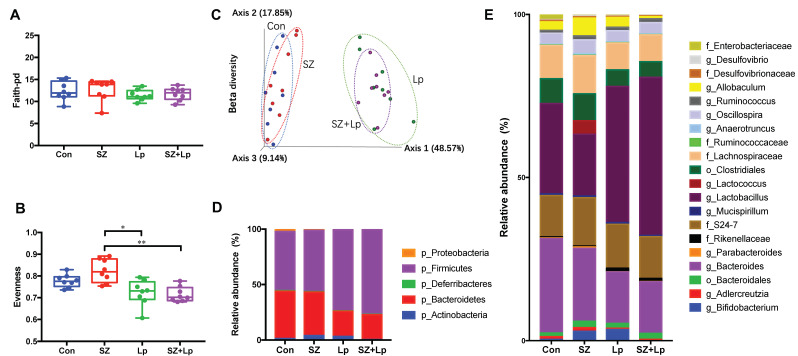
Comparison of diversity indices and microbial compositions amongst groups after 4 weeks of oral administration. Alpha-diversities of microbial communities are shown as (**A**) richness and (**B**) evenness. (**C**) Principal Coordinates Analysis (PCoA) plot represents beta-diversity based on Bray-Curtis dissimilarity. (**D**) Taxonomic profiles at the phylum level (**D**) and the genus level (**E**). Relative Abundance of taxa below 0.1% were excluded prior to analyses. Significance was accepted at * *p* < 0.05, ** *p* < 0.01 (*n* = 8). Con, control; SZ, selenium and zinc supplemented; Lp, *Lactobacillus plantarum* SeZi; SZ + Lp, selenium- and zinc-enriched *L. plantarum* SeZi.

Gut microbiota alpha diversities were assessed by richness (Faith-pd) and Pielou’s evenness analyses, which represent the number of species and the degree of species homogeneity, respectively. No significant difference was observed between the groups in the alpha diversity of richness (Figure 3A), while the evenness in the SZ group was significantly larger than Lp and SZ + Lp groups (Figure 3B). The results of beta diversity (Bray-Curtis dissimilarity) indicated that the clustering in microbial communities in the Lp and SZ + Lp group was distinct from that in the control and SZ group (Figure 3C).

**Figure 4:**
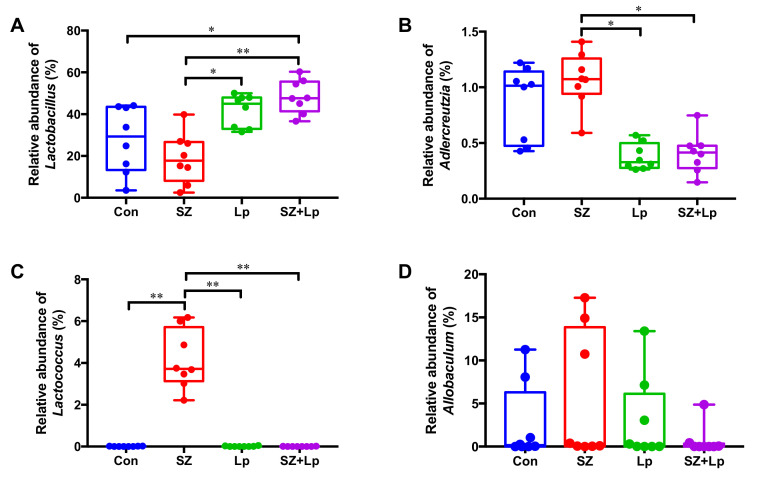
Relative abundances of *Lactobacillus* (**A**), *Adlercreutzia* (**B**), *Lactococcus* (**C**), and *Allobaculum* (**D**) in fecal samples after 4 weeks of oral administration. Data are expressed as mean ± SD. Significance was accepted at * *p* < 0.05, ** *p* < 0.01 (*n* = 8). Con, control; SZ, selenium and zinc supplemented; Lp, *Lactobacillus plantarum* SeZi; SZ + Lp, selenium- and zinc-enriched *L. plantarum* SeZi.

The average relative abundances of the final day fecal samples at the phylum level (Figure 3D) and the genus level (Figure 3E) suggested the different microbial compositions amongst the groups after the oral administration of SZ and SZ + Lp. To further evaluate the effects of treatments on microbial compositional changes, the three significantly different genera between the groups were identified and are displayed in Figure 4A–C. The relative abundance of *Lactobacillus* in the SZ + Lp group was significantly higher than that of the control and SZ groups, and the *Lactobacillus* level in the Lp group was also significantly higher than the SZ group. *Adlercreutzia* was significantly abundant in the SZ group compared to the SZ and SZ + Lp groups. Interestingly, *Lactococcus* was highly enriched only in the SZ group with the relative abundance at 4.15%. In addition, the relative abundance of *Allobaculum* in the SZ group was much larger than the SZ + Lp group, although the difference was not statistically significant (Figure 4D, *p* > 0.05).

### 3.5. Microbial Function Analysis Related to Selenocompounds Metabolism

To investigate the functional changes in selenocompounds metabolism of the gut microbiome, KEGG analysis was performed by phylogenetic investigation of the community by reconstruction of unobserved states (PICRUSt).

**Figure 5:**
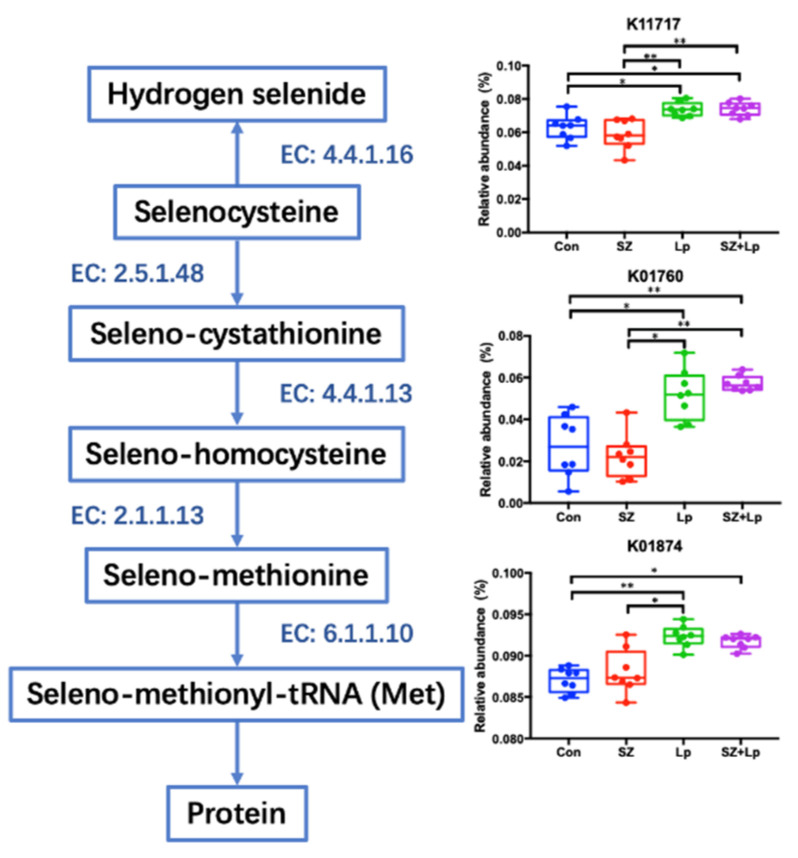
Selenocompounds metabolism pathway. Related enzymes include selenocysteine lyase (EC: 4.4.1.16), cystathionine gamma-synthase (EC: 2.5.1.48), cysteine-S-conjugate beta-lyase (EC: 4.4.1.13), homocysteine methyltransferase (EC: 2.1.1.13), and methionyl-tRNA synthetase (EC: 6.1.1.10). Arrows indicate the related genes that are involved in the corresponding pathway. Error bars represent means ± SD. Significance was accepted at * *p* < 0.05, ** *p* < 0.01 (*n* = 8). Con, control; SZ, selenium and zinc supplemented; Lp, *Lactobacillus plantarum* SeZi; SZ + Lp, selenium- and zinc-enriched *L. plantarum* SeZi. K11717, cysteine desulfurase/selenocysteine lyase; K01760, cystathionine beta-lyase; K01874, methionyl-tRNA synthetase.

As presented in Figure 5, *L. plantarum* SeZi significantly increased the relative abundances of the SCLY gene coding for selenocysteine lyase (EC: 4.4.1.16), CCBL gene coding for cysteine-S-conjugate beta-lyase (EC: 4.4.1.13), and MARS gene coding for methionyl-tRNA synthetase (EC: 6.1.1.10). These selenocompounds metabolism-related genes were responsible for the utilization of selenocysteine, seleno-cystathionine and seleno-methionine, respectively.

**Figure 6:**
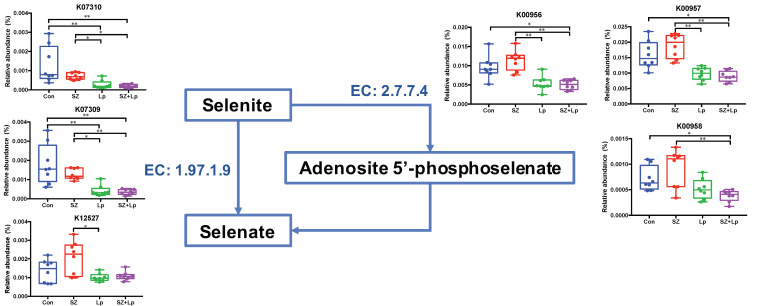
Detoxification process of inorganic selenium. Related enzymes include 3′-phosphoadenosine 5′-phosphosulfate synthase (EC: 2.7.7.4) and selenate reductase subunit alpha (EC: 1.97.1.9). Arrows indicate the related genes that are involved in the corresponding pathway. Error bars represent means ± SD. Significance was accepted at * *p* < 0.05, ** *p* < 0.01 (*n* = 8). Con, control; SZ, selenium and zinc supplemented; Lp, *Lactobacillus plantarum* SeZi; SZ + Lp, selenium- and zinc-enriched *L. plantarum* SeZi. K07310, Tat-targeted selenate reductase subunit YnfF; K07309, Tat-targeted selenate reductase subunit YnfE; K12527, putative selenate reductase; K00956, sulfate adenylyltransferase subunit 1; K00957, sulfate adenylyltransferase subunit 2; K00958, sulfate adenylyltransferase.

To detoxify selenite and selenate in the selenocompounds metabolism pathway, selenite is converted to selenate directly or via an intermediate, and selenate is further metabolized into hydrogen selenide. As shown in Figure 6, the relative abundances of enzymes related to oxidation of selenite to selenate (EC: 2.7.7.4, EC: 1.97.1.9) were significantly reduced in the Lp and SZ + Lp groups.

## 4. Discussion

In this study, *L. plantarum* SeZi isolate was screened out from human fecal bacteria by the selenium- and zinc-tolerant abilities. The selenium- and zinc-enriched *L. plantarum* SeZi strain increased the levels of selenium and zinc and presented antioxidative properties in an ICR mouse model. A thorough search of the literature reporting the bioavailability and functionality of Se- and Zn-enriched microorganisms using a mouse model yielded only one related article [42]. To determine appropriate concentrations of selenium and zinc for mice, we referenced this article and 300 μg of selenium and 1.5 mg of zinc (/kg body weight/day) were used in our study. Yan et al. reported the antioxidant and antitumor activities were significantly increased by supplementation with Se/Zn-enriched mushrooms. However, the in vivo antioxidant activity of Se/Zn-enriched LAB has not been reported yet.

Selenium is an essential element that must be exogenously provided to reach the requirement of human and animal health [43]. The toxicity order of selenium species from high to low is selenate, selenite, nano-selenium, and lactomicro-selenium [44]. The accumulation of selenium in bacteria is processed by extracellular binding via active groups in the cell-membrane conjunction or intracellular binding via ion transportation on the membrane [45]. Many *Lactobacillus* strains are well-known to accumulate and biotransform toxic selenite into non-toxic seleno-amino acids (i.e., selenocysteine and selenomethionine) and selenoprotein [46,47] The utilization of Se-enriched *Lactobacillus* possesses unique advantages, including low toxicity, low cost production and additional probiotic effects.

In this study, we focused on the changes in the gut microbial composition as well as functional metabolism associated with Se/Zn uptake and utilization. Interestingly, Se/Zn supplementation greatly induced the enrichment of specific genus, *Lactococcus* (belonging to LAB), which confirmed the previous reports regarding in vitro tolerance of LAB to selenium [46,47]. However, this indigenously enriched LAB by inorganic Se/Zn supplement showed significantly different patterns in the microbial selenocompounds metabolism compared with Se/Zn-bioaccumulated *L. plantarum* SeZi strain.

To further investigate strain-specific functions, whole genome sequencing of *L. plantarum* SeZi strain was conducted and analyzed based on public databases. The genes coding *cysA* and *dedA* were observed in the whole genome sequencing of *L. plantarum* SeZi. Both of the *cysA* and *dedA* genes are associated with selenite uptake and detoxification. These genes were abundantly present in *L. plantarum* SeZi strain, but not observed in *Lactococcus* or other *Lactobacillus* spp. According to previous studies, selenite may enter the cells of *E. coli* through the sulfate permease CysA [48]. DedA can uptake selenite into cells as a direct transporter or a cofactor. Additionally, the *dedA* gene-contained mutant *E. coli* displayed selenite resistance [17]. Based on the results of targeted metagenome sequencing, the *L. plantarum* SeZi strain promoted the utilization of selenocysteine, seleno-cystathionine and seleno-methionine in selenocompounds metabolism pathway of gut microbiome. Besides, the transformation between the toxic inorganic selenium was reduced by *L. plantarum* SeZi in the detoxification process. These are consistent with the selenium-related function detected in the genome sequencing of *L. plantarum* SeZi.

Zinc plays an essential role in catalytic, structural, and regulatory functions in enzymes and protein domains [49]. Similar to the other trace elements (i.e., selenium), zinc in the organic forms have more bioavailability its inorganic forms [49]. Pharmacological zinc supplements often have low bioavailability and are easily overdosed [50]. Although internalizing zinc by *Lactobacillus* has not been studied in depth, the utilization of certain *Lactobacillus* species can be a promising alternative to deliver zinc in a highly organic form [51]. 

The selenium and zinc levels in the blood can be affected by dietary supplementation and related metabolism. In this study, the concentrations of selenium and zinc in the Se/Zn-enriched *Lactobacillus* group were significantly higher than the control or SZ group. This is consistent with a recently published study which indicates that feeding a diet supplemented with Se/Zn-enriched probiotics, 0.3 mg/L selenium, and 100 mg/L zinc significantly enhanced the blood selenium and zinc concentrations in Wistar rats [52]. It is also the only published study referred to Se/Zn-enriched probiotics in a murine model to date. However, the assessment of antioxidant capability and its potential mechanisms was not reported yet.

In this study, the enhancement of antioxidant ability in the SZ + Lp group was probably triggered by the increased selenium level. When selenium is incorporated into the selenoenzymes (i.e., GSH-Px), it enhances the antioxidant activities by suppressing the nuclear factor-kappa B (NF-κB) signal pathway [53]. Most selenoproteins take part in the defense against oxidative stress, protecting tissues and cells from oxidative damages [47]. Although the SZ group was administered with inorganic forms of Se/Zn, the SZ + Lp group was administered with bio-accumulated Se/Zn in bacteria. To understand the mechanisms of bioavailability and functionality related to inorganic/organic forms of trace nutrients, it is necessary to investigate the changes in selenium and zinc metabolites (e.g., selenoproteins, zincproteins, etc.) from blood and fecal samples.

Alteration of microbial communities was evaluated by 16S rRNA community analysis in this study. Up to now, the 16S metagenomic technique is still rarely used in the selenium or zinc-related animal studies. Obviously, the significant surge of *Lactobacillus* levels in the Lp and SZ + Lp groups was caused by the oral administration of *L. plantarum* SeZi. The relative abundance of *Adlercreutzia* was significantly higher in the SZ + Lp group compared to the control and SZ groups. According to the previous studies, a decreased level of *Adlercreutzia* was observed in multiple sclerosis patients and Alzheimer’s disease patients [54,55]. Amongst *Adlercreutzia* species, *A. equolifaciens* is an equol-producing bacteria, promoting intestinal health [56]. Besides, a high relative abundance of *Lactococcus* (approximately 4.15%) was found in the SZ group, while this bacterium was almost undetectable in the other groups. It might be caused by utilization of selenium and zinc by *Lactococcus* spp. According to the previous studies, *Lactococcus lactis* is capable of selenium biotransformation and zinc uptake [57,58]. The relative abundance of *Allobaculum* spp., which was markedly reduced in the SZ + Lp group compared with other groups, was reported to be adversely associated with mRNA expression levels of tight junction protein genes (*zo-1* and *occludin*) and anti-inflammatory genes (*foxp3* and *Il-10*) in the colon of rats [59]. In some inflammatory bowel disease (IBD) patients, *Allobaculum* spp. was one of the uniquely observed species [60].

To the best of our knowledge, this study is the first attempt to evaluate the effects of Se/Zn-enriched LAB on in vivo gut microbiome changes. The microbiome analysis suggests a microbial aspect of selenocompound metabolism, however, lacks information on host’s Se/Zn metabolic process. Future studies on evaluating functionality of *L. plantarum* SeZi in a disease-induced mouse model should include host’s metabolites analysis as well.

## 5. Conclusions

In conclusion, the selected strain *L. plantarum* SeZi is able to resist and biotransform inorganic selenium into organic selenium. In the in vivo study, the selenium and zinc-enriched *L. plantarum* SeZi increased blood selenium level, antioxidant capability and the utilization of seleno-amino acids. Therefore, the *L. plantarum* SeZi strain is a potential selenium and zinc-enriched probiotic for application as functional food ingredients in the future.

## Figures and Tables

**Table 1:** Generation of biomass and bioconvertion rates of selenium and zinc in the selected SeZn-tolerant probiotic strains in vitro.

Strains	Biomass (g/L)	Bioconversion Rate (%)	
		Selenium	Zinc
*Lactobacillus plantarum* SeZi	2.82 ± 0.28 ^a^	19.47	0.35
*Lactobacillus pentosus* SeZi	2.33 ± 0.11 ^ab^	8.93	0.36
*Lactobacillus fermentum* SeZi	1.94 ± 0.34 ^bc^	18.04	0.33
*Lactobacillus rhamnosus* SeZi	1.78 ± 0.34 ^c^	6.90	0.20

Data are expressed as mean ± SD (*n* = 5). Treatments with different letters are significantly different at *p* < 0.05 (*n* = 5).

**Table 2:** Gene products of selenium resistance gene cluster in *Lactobacillus plantarum* SeZi.

Coding Region ^a^	Length (aa)	Product	Function
264974–265621 (−)	648	DedA protein	Detoxification and uptake of selenate
193218–193874 (−)	657	DedA protein	Detoxification and uptake of selenate
19823–20716	894	Sulfate permease-CysA	Detoxification and uptake of selenate

^a^ Genes encoded on the minus strand are indicated with (−).

**Table 3:** Gene products of zinc resistance gene cluster in *Lactobacillus plantarum* SeZi.

Coding Region ^a^	Length (aa)	Product	Function
237659–238102	444	ZUR	Zinc uptake regulation
151–966	816	Multidrug efflux transporter 1 regulator	Zinc resistance
31131–31565	435	Uncharacterized HTH-type transcriptional regulator	Zinc resistance
174455–174916	462	MerR family	Zinc resistance
22703–23146	444	MerR family	Zinc resistance
115660–116040	381	MerR family	Zinc resistance
27565–27957 (−)	393	MerR family	Zinc resistance
4022–4399	378	MerR family	Zinc resistance
227–619 (−)	393	MerR family	Zinc resistance
342254–342718	465	Hypothetical protein	Zinc resistance
54502–54756 (−)	255	Hypothetical protein	Zinc resistance
54351–54800	450	Hypothetical protein	Zinc resistance

^a^ Genes encoded on the minus strand are indicated with (−). ZUR: zinc uptake regulation.

**Table 4:** Other *Lactobacillus* strains with selenium and/or zinc resistance gene clusters. (From NCBI genome databases).

DedA	CysA	ZUR	MerR Family
*L. acidophilus* La-14	-	*L.rhamnosus* LOCK908	*L. acidophilus* La-14
*L. gasseri* ATCC 33323		*L.rhamnosus* LOCK900	*L. curvatus* JCM 1096
*L. dekbrueckii* subsp. *delbrueckii*		*L.rhamnosus* LOCK919	*L. gasseri* ATCC 33323
*L. salivarius* str. Ren			*L. ruminis* ATCC 25644
*L. buchneri* subsp. *silagei* CD034			*L. buchneri* subsp. *silagei* CD034
			*L. rhamnosus* GG
			*L. paracasei* subsp. *paracasei* 8700:2
